# Invited Review: “I’ve Just Seen a Face”

**DOI:** 10.1177/10556656241259885

**Published:** 2024-06-03

**Authors:** Kenny Ardouin

**Affiliations:** 1 School of Psychology, Speech & Hearing, 2496 University of Canterbury, Christchurch, New Zealand

**Keywords:** cleft lip and palate, parental perception, peers, social support, team care

## Abstract

I've Just Seen a Face is a new resource produced by author Amy Mendillo and is designed for parents of children with cleft lip and/or palate to help them to navigate the first year of life. In this invited article, Kenny Ardouin provides an overview of the book, and offers perspective on the content contained within, including potential issues for professionals working with cleft to consider. The review ends with recommendations for likely beneficiaries of the book, as well as considerations for additional future versions of the book.

As someone involved with the cleft community as both a clinician and person born with cleft, it has been encouraging to see new resources sharing stories of the community and which can empower new members of the community. Recently, I had the privilege of reviewing Amy Mendillo's new book *I’ve Just Seen a Face: A Practical and Emotional Guide for Parents of Children Born with Cleft Lip and Palate*.^1^ (Figure 1)

**Figure 1.**
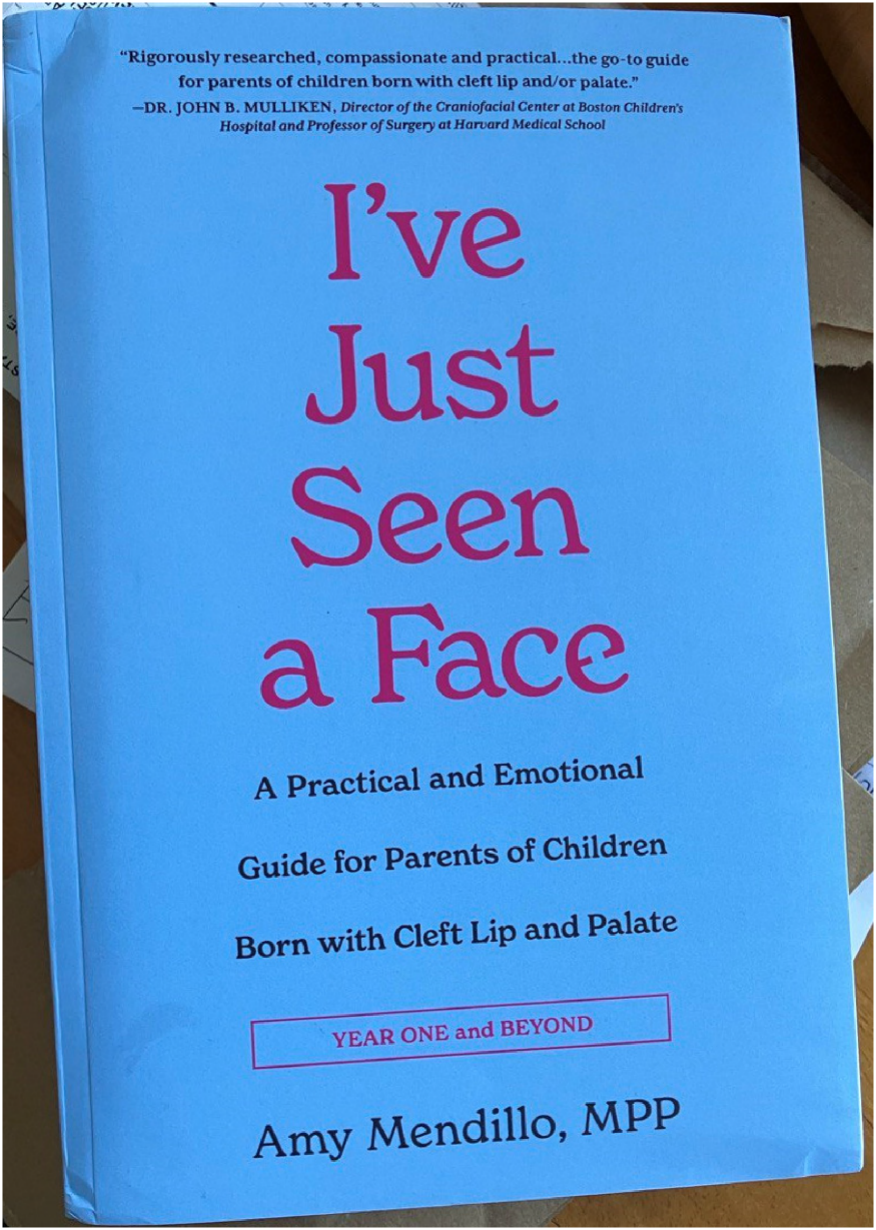
Cover of I’ve Just Seen a Face.

## Overview of the Book

This book is intended as a resource for parents who have just learned their child has, or will be born with, a cleft and provide a guide for navigating the first year of their child's life. *I’ve Just Seen a Face* would also be a book that parents could continually revisit during different stages of the cleft journey beyond the first year. Additionally, it is a fantastic resource to share with friends and family to raise early awareness and reduce the burden of continuously informing others about a child's cleft. The book takes a kind, gentle approach with simple explanations throughout, alongside sections of detailed information which makes it easy to dive deeper as desired.

The author is to be commended on the depth of information in this incredible piece of work – it is one of the most well-researched family-focussed resources of recent years. The author has interviewed almost 200 parents and professionals, often over multiple occasions and spanning a period of almost 10 years. The content is factually accurate, and Ms. Mendillo expertly transformed complicated concepts into explanations and terms that the typical (and potentially overwhelmed) parent will understand – a skill that those of us working as health professionals can take inspiration from.

In addition to the wealth of information, a strength of the book is its sensitivity in sharing different perspectives, views, life experiences, cultures, and faiths. The breadth of viewpoints creates a resource that is sure to resonate with people regardless of their experiences. Throughout, the author acknowledges there is no right or wrong way to feel about a child's cleft and that perspectives and feelings may change over time. The book acknowledges different emotions and reactions as the reality for individuals and affirms that these experiences are valid.

Within the narrative, clever switches between describing health information and sharing the experiences of various families affected by cleft (including the author's family's own story) balances subjective and objective truths associated with different cleft journeys. Ms. Mendillo has taken time to create a book by, for, and about the cleft community.

Although a sizeable book (over 500 pages), it is handily divided into 32 easy to read chapters which are thoughtfully organized and structured. An extensive reference list is evidence of the scientific rigor used in the creation of this publication, and readers will likely be grateful for the valuable glossary and index.

## Content Review of “I’ve Just Seen a Face”

### Cleft Basics

In addition to chapters, the book is separated into nine distinct sections, beginning helpfully with Cleft Basics, which succinctly discusses the fundamentals of cleft.

While the author has effectively curated information about the different services available in the US, the book also unwittingly draws attention to some of the practice differences in cleft care. For example, references to the reassurance that accessing fetal echocardiogram (ECG) can offer may be limited, and this presents some concern, knowing that lack of access could become a source of anxiety for parents where fetal ECG is not offered. As cleft providers, it will be important to address questions and the potential anxiety families might experience as they try to navigate their own experiences that may differ from what is described in the book.

Chapter four, *Why did this happen?,* provides a sensitive overview of how cleft occurs and continues to present scientific information alongside mindful acknowledgement of the philosophical, spiritual, and emotional reactions parents and family members can have to a cleft diagnosis.

### Preparing for Birth

The following two sections *Team Care* and *Birth* provide an excellent overview of the typical program of cleft care, and highlight the value of the team approach. Ms. Mendillo clearly describes the importance of seeking a full team rather than solely enlisting an enthusiastic local plastic surgeon. As a health professional, I appreciated the description of the planning, coordination, and discussions that occur behind the scenes. These are aspects of the multidisciplinary team approach that parents and patients do not tend to see, but are critical to delivery of evidence-based, holistic, patient-centered care. I hope it is reassuring for families to know that a team of people are discussing strategies and solutions together, rather than making decisions in isolation.

*I’ve Just Seen a Face* empowers readers through inclusion of tangible and otherwise challenging-to-find resources, such as a list of questions to ask prenatal health professionals. Many of the questions listed are likely questions parents would not otherwise know to ask, such as asking how often surgeons perform cleft procedures. As I read this section, I couldn’t help but contemplate how difficult and awkward some of these questions would be for many parents to ask a team. It struck me that perhaps as health professionals we can do more to proactively address some of these questions, for example by having greater transparency and using shared processes across cleft teams.

This section concludes with Chapter 11 *Birth and Bonding,* which is beautifully and honestly written. When viewing most cleft support websites, we are shown images and messages from people who bonded instantly to their child with a cleft and seldom hear stories of those who struggle with bonding in the early days and weeks. Bonding can be challenging for parents and this chapter validates these feelings – it was fantastic to see these experiences acknowledged so candidly.

### Feeding, Primary Surgeries, Clinic Visits, Supermarket Visits

The remainder of the book is packed with practical advice regarding various issues that typically present themselves within the first year. As a Speech Language Therapist, I was impressed by the accuracy of the information about feeding. There is much misinformation online about how to feed infants with cleft, but it was encouraging to see such accurate, detailed information presented here – this is certainly a resource I will recommend to new parents. Multiple feeding scenarios are discussed, which will offer reassurance to those experiencing challenges in their journey to feeding. Without judgment, the author diligently explains options around expressing/pumping milk some or all of the time alongside formula feeding options. My own approach is echoed by the variety of options presented - I wholeheartedly support the notion that parents be empowered to find their own rhythm and the feeding option that works best for them. This chapter is a good reminder that it is incumbent on all of us to support parents in their choices – these are not decisions taken lightly and judgmental providers add no benefit.

Chapter 17 *What to Say* offers practical advice on what to say to other people when *your* child has a cleft, and what to say to the parents of a child who has just been born with a cleft. In this chapter, Amy once again provides robust exploration of the emotions that surface during many interactions. I found myself nodding along and smiling as I recognized a few of the well-intentioned but ultimately unhelpful phrases such as “be grateful it's not worse!”

In Chapter 20 *The Clinic Visit*, I was disappointed to read that people still need to be prepared to have health professionals describe faces in the bluntest of terms. This is not to critique Ms. Mendillo's writing, for she handles it with her characteristic delicacy, but rather to outline my disappointment that such tactless descriptions are still common – I distinctly recall this pattern of insensitive language being used with me, and the surgeon saying “but I can say that, because I can fix it.” The notion that I was defective and needed to be “fixed” has stuck with me for many years. I hope rather than instructing parents to prepare their children for this ‘inevitability’, that we as providers can see a call to action to be more mindful of the vocabulary we are using.

The final few chapters of the book focus on the lip and palate surgeries themselves and provide another valuable resource, well worth revisiting in the days and months leading up to surgeries. The book details all kinds of practical suggestions to reduce anxiety on the day, such as knowing where to park the car and how to navigate within the hospital. It is fantastic to see a list of things to bring for a hospital stay – such lists can be incredibly reassuring. While these are overall a collection of excellent chapters, I feel Chapter 29 *Recovery at home* could offer some advice on what to do if something does go wrong during recovery – while rare, these events do happen and being prepared for things not going to plan can make us more resilient as we have some idea of what to do when things begin to go a bit awry.

### Beyond Year One

The book ends with a nod to the road ahead. Reflecting on my own journey, although cleft has always been there, there have been periods where cleft is more salient than others – I resonated with the way this chapter explores this. The author describes the importance of viewing life beyond cleft, taking time to really get to know your child and celebrate all the other aspects that make them who they are. This can help put cleft into perspective. It can feel in the early days that cleft becomes your whole world, but it will become just one facet of the person they grow to be. Soon enough, parents are certain to find they do not have a ‘cleft child’ – they have a child with their own identity, personality, interests, goals, and ambitions, with friends, hobbies, likes, and dislikes, with tantrums and joy, and wit and humor. Oh, and they happen to have been born with a cleft.

### Considerations for a Future Edition

*I’ve Just Seen a Face* is an excellent resource which I hope will be utilized by parents, families, and professionals working with cleft. As the book primarily focusses on the first year of life, I feel there is plenty of scope for a second volume to explore the remainder of the journey through childhood and adolescence.

In addition to a second volume, I can see significant benefit in an international version of this book. While non-US parents would certainly gain insight from the book, it is centered around the US cleft care system, which is fairly different to other systems, particularly with regards to healthcare funding. Additionally, the book discusses a range of treatment options which may not be routinely available, an especially important caveat for international readers. Similarly, the book places emphasis on selecting an appropriate cleft team for your child's care, which can be an option for some families within the US based on their insurance and location; however, in most international locations, cleft teams are determined based on geographic location.

Naturally, given the scope of *I’ve Just Seen a Face*, parent perspectives are shared throughout. Though excellent to have parent perspectives, there is still the opportunity to add additional insight from patients’ perspectives across their lifespans. Incorporating lived experience in this way may offer parents of newborns extra reassurance of the fulfilled lives that people who were born with cleft go on to live.

Finally, a potentially more accessible referencing style would be to have each page's references referred to in footnotes at the bottom of the page rather than in the lengthy references section at the end.

## Final Word

Amy Mendillo has created an excellent resource for anyone new to or unfamiliar with the world of cleft. She has done a stellar job of presenting complex scientific concepts in an informative and easy-to-follow fashion. I will be recommending *I’ve Just Seen a Face* to new parents of children with cleft, as well as to people who have cleft and wish to better understand their condition. I am also excited to use this book in clinical education as recommended reading for students who will go on to work with families and individuals with cleft. Kudos to Amy on her efforts in producing this exciting new resource.
